# Deciphering primate retinal aging at single-cell resolution

**DOI:** 10.1007/s13238-020-00791-x

**Published:** 2020-10-14

**Authors:** Si Wang, Yuxuan Zheng, Qingqing Li, Xiaojuan He, Ruotong Ren, Weiqi Zhang, Moshi Song, Huifang Hu, Feifei Liu, Guoqiang Sun, Shuhui Sun, Zunpeng Liu, Yang Yu, Piu Chan, Guo-Guang Zhao, Qi Zhou, Guang-Hui Liu, Fuchou Tang, Jing Qu

**Affiliations:** 1 State Key Laboratory of Membrane Biology, Institute of Zoology, Chinese Academy of Sciences, Beijing, 100101 China; 2 Beijing Advanced Innovation Center for Genomics, School of Life Sciences, Peking University, Beijing, 100871 China; 3 State Key Laboratory of Stem Cell and Reproductive Biology, Institute of Zoology, Chinese Academy of Sciences, Beijing, 100101 China; 4 University of Chinese Academy of Sciences, Beijing, 100049 China; 5 Advanced Innovation Center for Human Brain Protection and National Clinical Research Center for Geriatric Disorders, Xuanwu Hospital Capital Medical University, Beijing, 100053 China; 6 Institute for Stem Cell and Regeneration, CAS, Beijing, 100101 China; 7 Ministry of Education Key Laboratory of Cell Proliferation and Differentiation, Biomedical Pioneering Innovation Center, Beijing, 100871 China; 8 Peking-Tsinghua Center for Life Sciences, Academy for Advanced Interdisciplinary Studies, Peking University, Beijing, 100871 China; 9 National Laboratory of Biomacromolecules, CAS Center for Excellence in Biomacromolecules, Institute of Biophysics, Chinese Academy of Sciences, Beijing, 100101 China; 10 Disease Genomics and Individualized Medicine Laboratory, Beijing Institute of Genomics, Chinese Academy of Sciences, Beijing, 100101 China; 11 Department of Obstetrics and Gynecology, Center for Reproductive Medicine, Peking University Third Hospital, Beijing, 100191 China; 12 Stem Cell Research Center, Peking University Third Hospital, Beijing, 100191 China; 13 Department of Neurosurgery, Xuanwu Hospital, Capital Medical University, Beijing, 100053 China

**Dear Editor**,

The retina is a light-sensitive highly-organized tissue, which is vulnerable to aging and age-related retinal diseases. Specifically, progressive retinal degeneration leads to visual function deterioration and vision impairment in the elderly (Lin et al., 2016). In diseases such as age-related macular degeneration (AMD), retinitis pigmentosa (RP) and diabetic retinopathy (DR), pathological process lacking effective treatments profoundly and negatively impact on the quality of life in the elderly (Lin et al., 2016; Chen et al., 2019). Thus, an in-depth molecular assessment of the mechanisms driving retinal aging is of urgent scientific and medical importance.

The retina comprises a layered structure with neural retina and a layer of retinal pigment epithelium (RPE). The neural retina consists of elaborate neuronal circuitry for the detection of visual information whereas the RPE and adjacent choroid layer (a vascularized and pigmented connective tissue) provide nutrition and physical and metabolic support. The neural retina layer mainly contains three demarcated cellular populations, photoreceptors (PRs), interneurons and retinal ganglion cells (RGCs). PRs, including rod and cone photoreceptors (Rod and Cone for abbreviation), convert light into an electrical signal and then transfer it to interneurons (bipolar cells, for instance). Interneurons then transmit the signal to RGCs, whose axons coalesce into the optic nerve projecting to the visual cortex. In addition, the neural retina layer contains many other cells, for example, Müller glial cells (Müller), which serve as supporting cells for the neurons. With aging, the retina undergoes a series of alterations, including physiological, structural and functional alterations, reflected as morphologic changes and/or loss of PRs and RGCs, decreased Rod- and Cone-mediated responses to light, impaired RPE function, and immune dysregulation (Lin et al., 2016; Chen et al., 2019).

Due to ethical constraints and limited access to disease-free human retina tissues, in-depth studies of human retinal degeneration are not practically feasible. As a result, non-human primates (NHPs), including crab-eating macaques (*Macaca fascicularis*), have become a widely used primate model in retinal studies, as they share very similar genetic and physiological characteristics with humans (Li et al., 2020; Wang et al., 2020; Zhang et al., 2020). Most of the retinal cell types have been identified primarily by morphological criteria, which lacks the molecular resolution necessary for dissecting aging-related changes in the primate. With the advent of single-cell RNA sequencing (scRNA-seq) technologies, in-depth molecular surveys of cellular taxonomy and aging mechanism of primate ovary, aorta and pancreatic islet have been achieved (Li et al., 2020; Wang et al., 2020; Zhang et al., 2020). However, the effects of aging on retinal cell types including those present in neural retina and RPE, as well as cell types in choroid layer remain largely unknown.

Here, we established the single-cell transcriptomic atlas of the retina and adjacent choroid in young and aged NHPs, identifying 15 cell types with distinct gene expression signatures and finding several novel markers. Our analysis reveals that oxidative stress is a major aging feature of the cells in the neural retinal layer, whereas an enhanced inflammatory response is that of RPE and choroidal cells. We also found that the RPE cell is the cell type most susceptible to aging in retina, as evidenced by the decreased cell density as well as the highest numbers of differentially expressed genes (DEGs) overlapping with genes underlying aging and aging-related retinal diseases, along with aberrant cell-cell interactions with its two adjacent layers. Altogether, our study provides the roadmap for understanding retinal aging in a NHP model at single-cell resolution, enabling the identification of new diagnostic biomarkers and potential therapeutic targets for age-related human retinal disorders.

To investigate structural and molecular alterations during physiological aging, we obtained retinal and choroidal tissues from eight young and eight aged cynomolgus monkeys (Fig. S1A). A histological comparison revealed the width of the RPE-choroid layer, neural retinal layer and its internal retinal nerve fiber layer (RNFL), ganglion cell layer (GCL), inner plexiform layer (IPL), inner nuclear layer (INL), outer plexiform layer (OPL), outer nuclear layer (ONL), photoreceptor layer (PRL) remained largely unchanged in the aged retina, suggesting an overall structural stability of aged retina (Fig. 1A). However, the density of Cone and RPE cells was reduced in the aged retina, implying age-related impairment on retinal cells (Figs. 1B, S1B and S1C). Scanning electron microscopy (SEM)-based 3D reconstruction revealed a less compact structure between PRs and RPE in aged retina compared with that from young animals (Fig. 1C), indicating disconnected interactions between these two layers. In the aged RPE cells, we also observed aging-associated elevated lipofuscin level (Fig. 1D), an indicator of compromised retinal function due to disruption of digestion of photoreceptor outer segments (Kennedy et al., 1995). Together, these results demonstrate that the PR and RPE undergo compromised connections and disrupted function during physiological aging.

**Figure 1:**
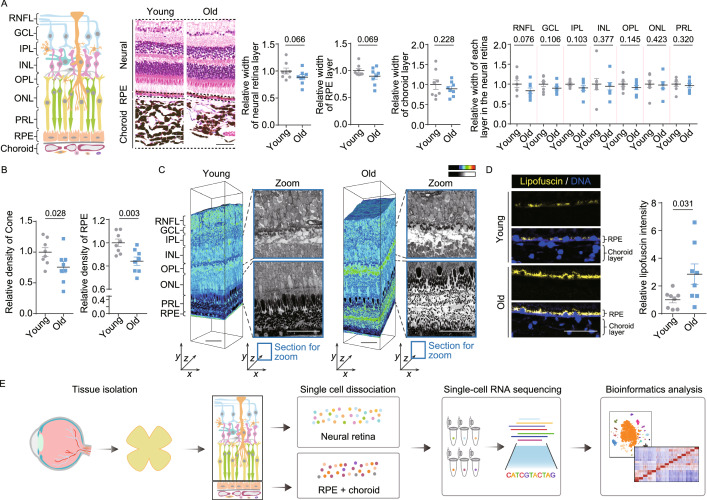

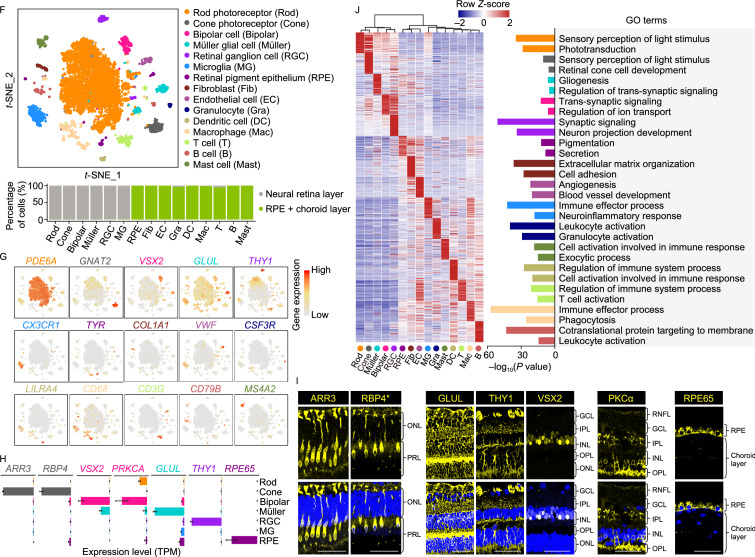
**Establishment of cynomolgus monkey single-cell transcriptome landscape of retinal and choroidal cells**. (A) Left, diagram showing the histological structure of monkey retina and choroid. Middle, representative images of H&E-stained sections of retinal and choroidal tissues from young and old monkeys. Right, the quantitative data for the relative width of each layer are shown as the mean ± SEM. Scale bar, 50 μm. Young, *n* = 8 monkeys; old, *n* = 8 monkeys. One-tailed student’s *t*-test *P* values are indicated. (B) The relative density of Cone and RPE cells are shown as the mean ± SEM. Young, *n* = 8 monkeys; old, *n* = 8 monkeys. One-tailed student’s *t*-test *P* values are indicated. (C) Large-scale three-dimensional reconstruction of a rectangular piece of retina using automatic collector of ultrathin sections scanning electron microscopy (AutoCUTS-SEM). Left, volume electron microscopy; right, the zoomed sections selected from the position of coordinate axes labeled with blue rectangles; the enlarged areas are indicated by black dash lines and shown without coloring with Imaris9.2.1 (color-key). Scale bar, 50 μm. (D) Representative images of lipofuscin accumulation in RPE are shown on the left, and quantitative data are shown as the mean ± SEM on the right. Scale bar, 50 μm. Young, *n* = 8 monkeys; old, *n* = 8 monkeys. Two-tailed Student’s *t*-test *P* value is indicated. (E) Study flowchart of single-cell RNA-seq in this study. (F) Top, *t*-SNE plot showing different cell types. Rod, rod photoreceptors; Cone, cone photoreceptors; Bipolar, bipolar cells; Müller, Müller glial cells; RGC, retinal ganglion cells; MG, microglia; RPE, retinal pigment epithelium; Fib, fibroblasts; EC, endothelial cells; Gra, granulocyte; DC, dendritic cells; Mac, macrophages; T, T cells; B, B cells; Mast, mast cells. Bottom, stacked bar plot showing the cell type distribution in the neural retina layer, RPE-choroid layer. (G) *t-*SNE plots showing gene expression signatures of representative marker genes for different cell types. The color key from grey to red indicates low to high gene expression levels. (H) Bar plots showing the expression level of representative marker genes for various cell types. All expression levels are measured using the same scale. Data are shown as mean ± SEM. (I) Immunofluorescence analysis showing specific expression of RBP4 and ARR3 in monkey Cone, THY1 in RGC, GLUL in Müller, VSX2 and PKCα in Bipolar, as well as RPE65 in RPE cells. Scale bar, 50 μm. Asterisk represents the newly identified marker. (J) Heatmap showing the top-ranked 100 (ranked by average difference among different cell types) gene expression signatures of each cell type. Each column indicates the mean expression level of the corresponding cell type, and values in each row are *z*-score scaled. Low to high gene expression levels are indicated by color key from blue to red. Representative GO terms are shown to the right

To dissect cell-type-specific aging-related gene expression changes, we utilized a modified single-cell tagged reverse transcription sequencing (STRT-seq) technique (Li et al., 2020; Wang et al., 2020; Zhang et al., 2020). First, we dissected the neural retina and RPE-choroid layers from eight young and eight aged animals, and subjected tissue samples to digestion and subsequent scRNA-seq (Fig. 1E). After stringent cell filtration, we retained 6,410 qualified single cells from 16 individuals for the downstream analyses (Fig. S1A and S1D; Table S1). Global gene expression profiling displayed no apparent differences in cell type distribution among monkeys of different ages and sexes, alleviating concerns about technical variations between samples (Fig. S1E). Unsupervised clustering analysis and *t*-distributed stochastic neighbor embedding (*t*-SNE) dimensionality analysis identified 15 distinct cell types characterized by their unique gene-expression signatures (Table S2). These included Rod and Cone, bipolar cells (Bipolar), RGC, Müller, and microglia (MG) in the neural retinal layer, RPE cells, as well as fibroblasts (Fib), endothelial cells (EC), granulocytes (Gra), dendritic cells (DC), macrophages (Mac), T cells (T), B cells (B) and mast cells (Mast) in the choroid layer (Fig. 1F). We mapped gene expression profiles of well-defined markers as shown in *t*-SNE plots and bar plots, including *PDE6A* for Rod, *GNAT2* and *ARR3* for Cone, *VSX2* and *PRKCΑ* for Bipolar, *GLUL* for Müller, *THY1* and *NEFM* for RGC, *CX3CR1* for MG, *TYR* and *RPE65* for RPE cells, as well as a panel of known markers for immune cells and other supporting cells (Figs. 1G, 1H and S1F). Of particular interest, we identified a set of novel markers of retinal cells, such as *AGRP* and *TFPI2* for Rod, *RBP4* and *NPTX1* for Cone, *TMEM37* and *SRGAP1* for Müller, *ATP1B1* and *RTN1* for RGC, as well as *SCGB3A2* and *TPM2* for RPE cells (Figs. 1H and S1F; Table S2). RBP4 was further validated by immunofluorescence staining specific to Cone (Fig. 1I). Gene Ontology (GO) analysis of cell-type-specific markers supported the identity of diverse cell types, revealing functional characteristics of NHP retinal and choroidal cells (Fig. 1J). For example, “sensory perception of light stimulus” for Rod and Cone, “gliogenesis” for Müller, “synaptic signaling” for RGC, “pigmentation” for RPE. In addition, our detailed analysis of Müller revealed a novel Müller glial cell subtype I (Fig. S2A and S2B; Table S2), with neuronal differentiation potential as indicated by subtype-specific DEG identification and GO enrichment analysis. Trajectory analysis ordered Müller glial cell subtype I at the beginning of the pseudotime trajectory (Fig. S2C), suggesting that Müller glial cell subtype I may have developmental potential to differentiate into other neural retinal cells. In addition, higher expression levels of several retinal progenitor markers, such as *ID3*, *SOX9* and *ZFP36L2* were also observed in subtype I (Lu et al., 2020), indicative of progenitor characteristics (Fig. S2D). Next, we performed a cross-species analysis among human, monkey and mouse to investigate cell identity similarities across species. As expected, the result indicates that the monkey retina recapitulates the human single-cell retinal transcriptional profile than mouse, as indicated by a higher correlation coefficient (Fig. S2E).

We next sought to explore cell-type-specific gene expression alterations associated with aging. We found similar transcriptional signatures of marker genes for each cell type between young and old monkey (Fig. S3A), demonstrating that aging had minimal effect on cell identity. Calculation of age-associated coefficient of variation (CV) demonstrated that Rod in the neural layer and Mast in the choroid layer exhibited higher transcriptional noise reflected as higher expression variations among individual aged cells than among non-aged cells (Fig. S3B). We noticed that the hotspot genes annotated in the GenAge gene set and gene sets implicated in retinal diseases including AMD and RP (Table S3) were highly expressed in Rod, Cone, RGC, RPE cells and Bipolar (Fig. S4), suggesting that these cell types may be implicated in retinal homeostasis and aging.

Based on analysis of aging-associated DEGs across all retinal and choroidal cell types, we found the largest numbers of upregulated DEGs in the RPE cells, EC and RGC (1,970, 1,561 and 1,560, respectively) in aged tissues, while the most downregulated DEGs were found in Gra and B cell types (939 and 840) relative to other cell types in the aged tissues (Fig. 2A; Table S4), indicating that these cell populations are most strongly affected by aging. Through GO analysis, we noticed that increased “response to oxidative stress” and “apoptotic signaling pathway”, as well as decreased “sensory organ development”, “transcription-coupled nucleotide-excision repair” and “visual perception” were enriched for DEGs of cells in the neural retinal layer (Fig. 2B; Table S4), indicative of elevated responses to external stresses and impaired functions in the aged neural layer. In parallel, GO analysis of DEGs in cells from the RPE-choroid layer revealed increased “cell activation involved in immune response” and “autophagy”, along with decreased “regulation of mitotic cell cycle” and “regulation of cytoskeleton organization” (Fig. 2B; Table S4), demonstrating age-related elevated inflammation and compromised supporting abilities in aged ﻿RPE-choroid layer. Generally, senescent cells are known to contribute to aging and age-related diseases by generating a low-grade inflammatory state (senescence-associated secretory phenotype, SASP). Indeed, when we enumerated the senescent state of various cell types in aged tissue, we found higher SASP score in most aged cells, especially in the RPE cells and cells from choroid layer (Figs. 2C, S5A and S5B; Table S3). Next, we performed a joint comparative analysis of aging-associated DEGs and genes from the GenAge gene set, or from the retinal disease-associated gene sets to identify the high-risk DEGs for retinal degeneration (Fig. 2D). This analysis highlighted potentially important regulators among the upregulated aging-associated DEGs, including *EGFR*, *AGBL5*, *STAT3* (Fig. 2D). For instance, increased *EGFR* expression may lead to disruption of tight junctions in RPE and diabetic retinopathy progression (Sugimoto et al., 2013), while *AGBL5*, a metallocarboxypeptidase involved in antiviral immunity and *STAT3*, a crucial transcription factor essential for inflammation response (Hutchins et al., 2013), are factors whose upregulation may augment inflammation and exacerbate pathological processes in aged retinal tissues. The potentially important regulators we identified as downregulated aging-associated DEGs included *TCF3*, *HDAC2* (Fig. 2D). TCF3 is associated with retinitis pigmentosa by forming HAND1::TCF3 complex and regulating the expression of retinal degeneration-related genes (Kaukonen et al., 2020). HDAC2 is one of histone deacetylase, whose downregulation is implicated in the pathogenesis of glaucoma by epigenetic regulation of extracellular matrix (ECM) genes (Park et al., 2017). Thus, downregulation of these broad-acting transcriptional regulators may result in disturbed epigenetic/transcriptional regulation networks, which by feeding into aberrant downstream cascades may contribute to retinal aging and related diseases in the aged retina. Moreover, joint comparative analysis pinpointed that the RPE cells and RGC were the cell types most affected by aging and retinal diseases in the retinal layers, as evidenced by the most enriched overlapping genes (Figs. S6, S7A and S7B). For example, multiple high-risk genes critical for AMD pathogenesis, including apolipoprotein E (*APOE*), vascular endothelial growth factor A (*VEGFA*) and tetraspanin 10 (*TSPAN10*) were upregulated in the aged RPE cells (Fig. S7A and S7B).

**Figure 2:**
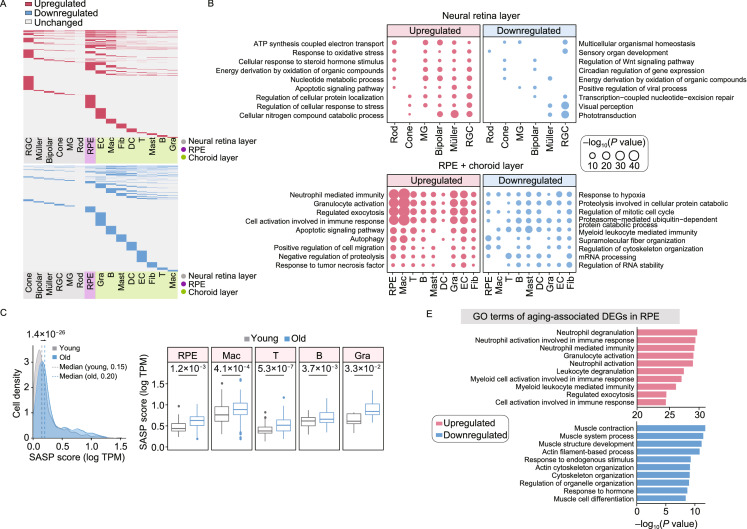

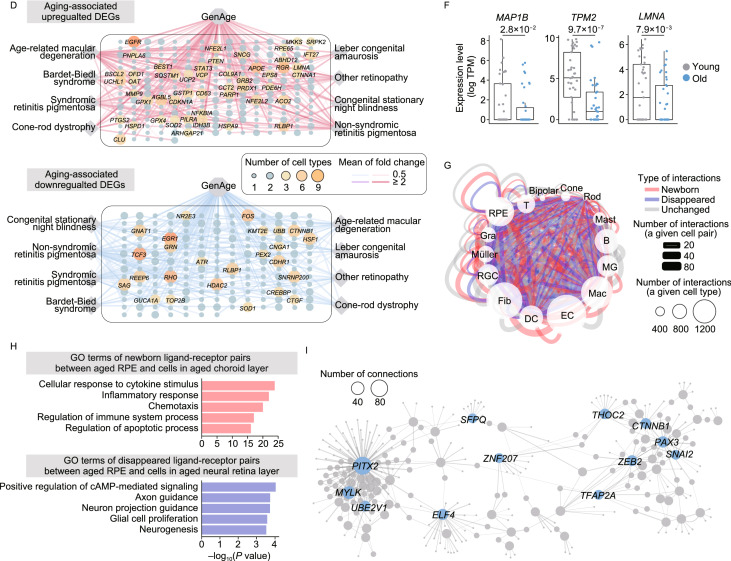
**Age-related transcriptional alterations in various cell types of monkey retina and choroid**. (A) Heatmaps showing upregulated (top) and downregulated (bottom) aging-associated DEGs in different cell types. DEGs present in at least two cell types are top-ranked in each panel. The shadow at the bottom of each panel indicates the layer corresponding to each cell type. (B) Dot plots showing GO terms of aging-associated DEGs in each cell type. Top, cell types from the neural retinal layer; bottom, cell types from the RPE-choroid layer. The dot size indicates the statistical significance of a corresponding GO term. (C) Left, density plot showing the cell distribution with different SASP scores in both young and old monkeys. Cells in all different cell types are considered. Grey and blue colors correspond to cells from young and old monkeys, respectively. Dashed lines indicate the median value of SASP score in cells from young (0.15) and old (0.20) monkeys, respectively; two-tailed Student’s *t*-test *P* value (1.4 × 10^−26^) is indicated. Right, box plots showing SASP score in representative cell types including RPE cells and cells in choroid layer. Two-tailed Student’s *t*-test *P* values are indicated. SASP score is calculated as the average expression level of SASP-related genes in each cell. (D) Network visualization showing the overlapping genes between aging-associated DEGs (top, upregulated; bottom, downregulated) and given gene sets (including GenAge and retinal disease gene sets). Each node in this network indicates each gene, and each line indicates the correspondence between a given gene set and a given DEG. The node size and color indicate the number of cell types in which a given gene was differentially expressed during aging. The line thickness and color indicate the average value of log_2_-transformed fold change of corresponding cell type(s). Gene names with node size greater than three are shown. (E) Bar plots showing GO terms of upregulated (top) and downregulated (bottom) aging-associated DEGs in RPE cells. (F) Box plots showing the expression level of representative downregulated aging-associated DEGs of RPE cells. Each point represents a single cell. *P* values obtained from ROTS algorithm are indicated. (G) Network visualization showing cell-cell interactions among different cell types. The line color indicates the type of interactions; red, newborn interactions (only existed in aged monkeys); blue, disappeared interactions (only existed in young monkeys); grey, unchanged interactions (commonly existed in young and old monkeys). The line thickness indicates the number of interactions between a given cell pair, and the dot size indicates the number of interactions in a given cell type. (H) Bar plots showing GO terms of newborn ligand-receptor gene pairs between aged RPE cells and cells in aged choroid layer (top), and disappeared ligand-receptor gene pairs between aged RPE cells and cells in aged neural retina layer (bottom). (I) Regulatory network showing potentially core transcriptional regulators in downregulated aging-associated DEGs of RPE cells. The line thickness indicates the weight of a connection, and the dot size indicates the number of connections. Gene names of top-ranked 10 nodes (ranked by the number of connections) are shown

As the core epithelial sheet interfacing the neural and choroid layer, RPE cells display strong apical (towards photoreceptors) to basal/basolateral (towards Bruch membrane) polarization which is maintained by the cytoskeleton, highly organized and polarized intracellular structure (Tarau et al., 2019). We found that a panel of downregulated aging-associated DEGs were enriched for “cytoskeleton organization” in aged RPE cells (Fig. 2E), including *MAP1B* [log_2_(fold change) = −0.67, *P* value = 2.8 × 10^−2^], encoding a specific marker protein of the podocyte microtubular cytoskeleton, *TPM2* [log_2_(fold change) = −3.01, *P* value = 9.7 × 10^−7^], encoding beta-tropomyosin, a member of the actin filament binding protein family, as well as *LMNA* [log_2_(fold change) = −0.92, *P* value = 7.9 × 10^−3^] (Fig. 2F), encoding a nuclear lamina protein. Their downregulation may contribute to age-related aberrant cytoskeleton organization and depolarization in aged RPE cells, and compromise the signaling communications between its two adjacent layers. When we performed cell-cell interaction analysis across all cell types (Fig. 2G), we found that global cell-cell interactions were enhanced in the aged RPE cells (1,029 interaction pairs in old tissues versus 766 pairs in its young counterparts) (Fig. S8; Table S5). Specifically, the disappeared ligand-receptor pairs between aged RPE cells and cells in neural layers were enriched in “axon guidance” and “neuron projection guidance” (*BMPR1B-BMPR2*) pathways (Figs. 2H and S8), suggesting that dysregulated cell-cell interactions impair the RPE-photoreceptor communications and result in loss of retinal homeostasis in aged retinal tissues. The newborn ligand-receptor pairs between aged RPE cells and cells in the choroid layer, enriched in the “inflammatory response” and “chemotaxis”. The representative pairs were “*CCL8*-*CCR3*” and “*IL34*-*CSF1R*” (Figs. 2H and S8), capable of destabilizing the retinal and choroidal microenvironment by inducing chronic inflammation, ultimately accelerating retinal dysfunction and vision impairment (Chen et al., 2019). When we constructed transcriptional regulatory networks to explore master regulators of aging-associated DEGs in different cell types (Figs. 2I, S9A and S9B), we identified several downregulated transcriptional regulators modulating aging-associated DEGs in RPE cells, such as *PITX2* and *CTNNB1* (Fig. 2I), both of which are involved in the Wnt/beta-catenin pathway. Since Wnt/beta-catenin signaling is vital for RPE proliferation after damage (Han et al., 2015), its dysregulation is likely to dampen RPE repair ability and accelerate retinal aging in the elders.

In this study, we generated the first primate single-cell transcriptome atlas of retinal aging including comprehensive cellular and molecular signatures in both the neural retina and the RPE-choroid layer. By delineating unique gene-expression profiles, we annotated 15 different cell types and identified several novel markers. Aging-associated DEG analysis revealed layer-specific gene expression alterations associated with increased stress response and compromised sensory and neural function in the neural retina layer, and an elevated inflammatory response and diminished supporting roles in the RPE-choroid layer. Cumulatively, our analyses underscore an important role of the RPE and its enhanced inflammatory response and depolarization may contribute to RPE functional decline with age, and function as a conduit for triggering the formation of an inflammaging microenvironment in the retina.

The retina is an attractive model to investigate fundamental biology and is of strong therapeutic interest. However, due to ethical issues, obtaining age-matched healthy human retinal tissues is difficult. Most importantly, due to the fact that aging is both a slow biological process and heterogeneous across individuals, modeling physiological aging is extremely difficult. Therefore, we lack a fundamental understanding of the cell-type-specific molecular mechanisms that trigger and exacerbate retinal aging and aging-related retinal degenerative diseases. With advances of scRNA-seq technologies, it has become possible to accurately delineate molecular features of the aging process by time-series experiments at the single-cell level (Li et al., 2020; Wang et al., 2020; Zhang et al., 2020; Zheng et al., 2020). We here delineate a primate single-cell transcriptome landscape of retinal aging, which depicts the detailed analysis of the effect aging has on diverse cell types in the neural retina and the RPE-choroid layer based on the high-quality and precise transcriptomic data using a modified STRT-seq technique (Li et al., 2020; Wang et al., 2020; Zhang et al., 2020). Of note, we characterized a Müller glial cell subtype that resembles retinal progenitor cells (RPCs) with uniquely elevated levels of a panel of progenitor cell markers. Consistent with our data, a recent report proposing that Müller glial cells emerge from a subset of RPCs, and a number of the genes upregulated in Müller glial cells begin their expression in RPCs during retinogenesis (Lu et al., 2020). In the adult retina, Müller glial cells constitute a potential source for regeneration after exposure to neurotoxic injury (Ooto et al., 2004), pointing to the possibility that this cell population can be targeted in retina regeneration. However, the amacrine cells (ACs) and horizontal cells (HCs) were not identified in our study. This may be explained by the possible bias introduced by the technical challenges of obtaining these cells with current tissue digestion and/or manually picking methods, which might be overcome via spatiotemporal transcriptomic analysis of retinal tissues in future studies.

Retinal cells encounter a large amount of light, making them vulnerable to light-induced damage (Lin et al., 2016). Accordingly, we found that aging-associated DEGs were enriched for external stress and apoptosis pathways in the neural layer. Interestingly, we also observed impaired transcription-coupled nucleotide-excision repair in the aged neural layer, which may result in accumulated light-induced DNA damage, compounding the loss of neural cells in the aged retina. At the retinal/choroidal interface, RPE is vital for maintaining homeostasis of the RPE-photoreceptor interactions, as well as supporting the structure and function of the choroidal microenvironment. Given that inflammation is an adaptive response to noxious stress or malfunction, the elevated immune response and inflammation we observed in the aged RPE cells and cells in the choroid layer, implies that the retinal niche balance has become skewed towards a chronic stress state. Consistently, we noticed preferential age-related accumulation of lipofuscin in the aged RPE. A recent study has suggested that various components of heterogeneous lipofuscin deposits may drive immune dysregulation via monocyte and microglial activation (Lin et al., 2016), ultimately leading to photoreceptor death (Kennedy et al., 1995). We also found that cytoskeleton organization was impaired in aged monkey RPE, a hallmark feature of many retinal diseases, including AMD (Tarau et al., 2019). Altogether, our data identified a serial of underlying molecular processes subjecting the primate retina to external and internal stresses during physiological aging, many of which may contribute to age-related retinal degeneration.

In conclusion, we establish a precise single-cell transcriptomic landscape of primate retinal aging, which provides deeper understanding of diverse mechanisms underlying retinal aging in primates. Our work also serves as a rich resource for identifying potential biomarkers for earlier clinical diagnosis of retinal aging-associated diseases and targets for development of novel therapeutic interventions to treat aging-related retinal diseases.

## Electronic supplementary material

Below is the link to the electronic supplementary material.

Supplementary material 1 (PDF 2328 kb)

Supplementary material 2 (XLSX 606 kb)

Supplementary material 3 (XLSX 645 kb)

Supplementary material 4 (XLSX 27 kb)

Supplementary material 5 (XLSX 13780 kb)

Supplementary material 6 (XLSX 907 kb)

Supplementary material 7 (XLS 27 kb)
