# Supplementary material for: Deciphering primate retinal aging at single-cell resolution
Source: Protein Cell. 2020 Oct 14;12(11):889–98. doi: 10.1007/s13238-020-00791-x (PMC8563949; doi:10.1007/s13238-020-00791-x)
Supplement: Supplementary file 1 — Supplementary material 1 (PDF 2328 kb) [file 13238_2020_791_MOESM1_ESM.pdf]

Materials and Methods

Ethical statement

This study was conducted following the guidelines for the Ethical Treatment of Non-Human Primates and was approved by the Institutional Animal Care and Use Committee of the Institute of Zoology (Chinese Academy of Sciences).

Experiment models and biological samples

All cynomolgus monkeys (*Macaca fascicularis*) were of Southeast Asian origin, and raised at around 25°C on a 12-hour (h) light, 12-h dark schedule at Xieerxin Biology Resource, an accredited primate research facility in Beijing. All animal experiment procedures were performed by certified veterinarians, complying with laws governing animal research. All animals were given commercial monkey diet twice a day and vegetables and fruits once a day with tap water *ad libitum* (Li et al., 2020; Wang et al., 2020; Zhang et al., 2020). None of the animals had a clinical or experimental history potentially affecting the physiological aging process. Retinal and choroidal tissues for scRNA-seq analysis were obtained from 8 young (4-6 years old) and 8 aged cynomolgus monkeys (18-21 years old).

Single cell isolation and collection

To ensure tissue quality, animals were sampled on different days. In brief, the animal was anesthetized and perfused with physiological saline, and the peripheral retinal and choroidal tissues were isolated. The neural retina layer and RPE-choroid layer were dissected and processed separately. Samples were minced and then transferred into 1.5 mL tubes and subjected to 1 mL of digestion buffer containing 1 mg/mL Collagenase, Type IV (Gibco 17104-019) and 1 mg/mL dispase II. After incubation at 37°C for 15 min on a shaking thermomixer (1000 rpm), tubes were briefly spun at 500 x *g* for 8 min at 4°C, and the supernatant was then gently removed. Pellets were resuspended in 1 mL PBS containing 10 % FBS and gently pipetted on ice for 50-100 times to dissociate the cells and the resulting supernatant immediately filtered through 100-μm mesh filters to remove undigested tissues. Debris was removed by fluorescence-activated cell sorting (FACS) (BD FACSAria™ II) and samples were subjected to single-cell collection. The purified cell suspension was then transferred into a 3.5 cm dish and cells were randomly picked by mouth pipette under a dissection microscope. For each animal, approximately 384 cells were picked for the neural retina layer, and 48-96 cells for the RPE-choroid layer. Each cell was picked into an individual PCR tube with lysis buffer and stored at -80°C for subsequent experiments.

Single-cell RNA-seq library preparation and sequencing

Single cell transcriptome amplification was performed with a modified STRT-seq protocol, as previously described (Dong et al., 2018; Islam et al., 2012). Briefly, single cells were randomly collected by a month pipette and transferred to lysis buffer. After cell lysis, released mRNAs were reverse transcribed into cDNA by barcode-reverse transcription primers with 8 bp unique molecular identifiers (UMIs). Then, the second-strand cDNAs were synthesized, followed by cDNA pre-amplification. The amplified cDNAs were pooled together and purified for fragmentation after which 3' ends of fragmented cDNAs were collected for library construction with Kapa Hyper Prep Kit. Then, sequencing was performed on HiSeq 4000 platform with 150 bp paired-end reads.

Hematoxylin and Eosin (H&E) staining

H&E staining was performed according to previous studies (Nichols et al., 2005; Wang et al., 2020; Zhang et al., 2020). The density of RPE cells was calculated by the number of RPE cells divided by the volume analyzed.

Large-scale three-dimensional retina reconstruction

Tissues were fixed with 2.5% (vol/vol) glutaraldehyde with Phosphate Buffer (PB) (0.1 M, pH 7.4), washed four times in PB. Then the samples were immersed in 1% (wt/vol) OsO4 and 1.5% (wt/vol) potassium ferricyanide aqueous solution at 4°C for 1 h. After several washes, the samples were incubated in filtered 1% thiocarbohydrazide aqueous solution (Sigma-Aldrich) at room temperature (RT) for 30 min, 1% unbuffered OsO4 aqueous solution at 4°C for 1 h and 1% UA aqueous solution at 4°C overnight following four rinses in ddH_2O for 10 min each between each step. Next, the samples were dehydrated through graded alcohol (30%, 50%, 70%, 80%, 90%, 100%, 100%, 10 min each step) at 4°C into pure acetone (3×10 min). Samples were infiltrated in a graded mixture (3:1, 1:1, 1:3) of acetone and SPI-PON812 resin (19.6 mL SPI-PON812, 6.6 mL DDSA and 13.8 mL NMA, 1.5% BDMA), then changed with pure resin. The samples were finally embedded in pure resin with 1.5% BDMA and polymerized for 12 h at 45°C, 48 h at 60°C.

Automatic collector of ultrathin sections scanning electron microscopy (AutoCUTS-SEM) was performed as previously described (Li et al., 2017; Zhang et al., 2020). In brief, about 2000 sections were collected by the ultramicrotome (UC7, Leica, Germany) with the AutoCUTS device for each sample. Next, high throughput serial sections were automatically acquired by a Helios Nanolab 600i dual-beam SEM (Scanning Electron Microscope, FEI) with an automated software (AutoSEE), and an image reconstruction program was conducted. The image parameters including accelerating voltage of 2 kV, beam current of 0.69 nA, CBS detector, pixel size of 58.6 nm and dwell time of 5 μs. About 988 and 1121 sections were collected for young and old samples, respectively.

Tissue immunostaining

Immunostaining was performed following the previously published studies (Ma et al., 2020; Wang et al., 2020; Zhang et al., 2020). First, monkey retinal and choroidal tissues were fixed with 4% paraformaldehyde (PFA) at 4°C overnight, soaked in 30% sucrose, embedded in Tissue-Tek® O.C.T.™ Compound (Sakura Finetek, 4583) and frozen. Frozen sections (10 μm) were obtained and stored at -80°C prior to use. Antigen retrieval was performed by microwaving the sections at 98°C in 10 mM sodium citrate buffer (pH 6.0) for three times (5 min each time). After cooling down, the slides were washed three times with PBS, permeabilized with Triton X-100 (0.4% in PBS) for 25 min, blocked with 10% donkey serum in PBS for 1 h at RT, and stained with primary antibodies at 4°C overnight. Then, after washes with PBS, sections were incubated with secondary antibodies for 1 h at RT. Nuclear DNA was stained by Hoechst 33342 (Thermo Fisher Scientific). Next, sections were mounted with VECTASHIELD® Antifade Mounting Medium (Vector Laboratories, H-1000), and fluorescent images were obtained using a confocal microscope (Leica TCS SP5 Ⅱ). The antibodies used in this study are shown in Supplementary material, Table S6. The density of Cone was calculated by the number of ARR3-positive Cone divided by the volume analyzed.

Lipofuscin fluorescence imaging

Lipofuscin fluorescence imaging was performed as previously described with minor modification (Dorey et al., 1989). The paraffin section was deparaffinized and

permeabilized with Triton X-100 (0.4% in PBS) for 25 min, stained with Hoechst 33342 and mounted with VECTASHIELD® Antifade Mounting Medium. Next, the fluorescence imaging was performed by using a confocal microscope (Leica TCS SP5 Ⅱ).

Single-cell RNA-seq data processing

Raw paired scRNA-seq data in each library was split into single cells based on barcode sequences. To obtain clean reads for each cell, the scRNA-seq data was first processed by trimming the template switch oligo (TSO) and poly(A) tail sequences, and then removing the reads with adapters and low-quality bases. Next, clean reads were mapped to the *Macaca fascicularis* reference genome (Ensembl version: Macaca_fascicularis_5.0) using *Tophat* (version: 2.0.12) (Trapnell et al., 2009). UMIs within the uniquely mapped reads were counted for each gene using *HTSeq* (Anders et al., 2015), in which process the same UMI sequences were only counted once.

We quantified the gene expression level with transcripts per million (TPM) which was calculated as the number of UMIs of a given gene in a given cell divided by the total number of UMIs of this given cell and then multiplied by 1,000,000. Gene expression levels were then transformed into $\log_2(\mathrm{TPM}/10 + 1)$ since the scRNA-seq library complexity was estimated to be ~100,000 transcripts.

To obtain high-quality single cells for the downstream analysis, cells that met the following three criteria were retained: the rate of uniquely mapped reads aligned to the reference genome was greater than 10%, the number of detected genes was greater than 800, and the number of detected UMIs was greater than 5,000 (Fig. S1C; Table S1). After this quality filtering process, 6,410 out of 7,461 cells were retained for further analysis. In high-quality single cells, the median rate of mapped reads was 41.8%, and the median number of detected genes and UMIs was 2,439 and 29,869, respectively.

Identification of cell types and cell-type-specific marker genes

Based on the transformed TPM data, 1,126 highly variable genes (HVGs) were identified using R package *Seurat* (Satija et al., 2015) with parameters '*x.low.cutoff = 1, x.high.cutoff = 10, y.cutoff = 1*' according to the average expression and dispersion, and then these selected HVGs were used to perform principle component analysis (PCA). To exclude the principal components (PCs) which explained very little of variance and improve the signal-to-noise ratio as described in the previous study (Haber et al., 2017), 'significant' components were chosen using function *JackStraw* in *Seurat* based on the permutation test, and 30 significant components (PC1~PC30) were used for *t*-distributed stochastic neighborhood embedding (*t*-SNE) analysis. Unsupervised clustering analysis was performed using function *FindClusters* in *Seurat* with the parameter '*resolution = 2*', and different clusters were labeled by the expression levels of known marker genes.

A standard area under the curve (AUC) classifier was utilized to identify cell-type-specific markers (differentially expressed genes (DEGs) among different cell types) with function *FindAllMarkers* in *Seurat*. Marker genes were chosen only if the average difference of the $\log_2$-transformed TPM was greater than 1 with a statistical significance *power* greater than 0.25. Gene ontology (GO) analysis of cell-type-specific marker genes was performed using website tool *Toppgene* with default parameters (Chen et al., 2009).

Pseudotime trajectory analysis of the subpopulation of Müller glial cells

Based on scRNA-seq data in which gene expression levels were quantified with raw

UMI count data, pseudotime trajectory analysis was performed using R package *monocle* (version: 2.10.1) (Qiu et al., 2017; Trapnell et al., 2014). To exclude effects from other cells, only specific retinal cell types were considered, including Müller, Rod, Cone, Bipolar and RGC, comprising 4,946 cells in total. HVGs were computed based on the average expression and dispersion using function *dispersionTable* in *monocle* with default parameters, and 3,057 HVGs were selected with filtering condition '*mean_expression >= 0.01 & dispersion_empirical >= 1.5 * dispersion_fit*'. To ensure reproducibility, the random seed was set as '*19961109*' in this analysis, and functions *reduceDimension* and *orderCells* were used to compute a projection of cells into a lower dimensional space and order cells according to pseudotime, respectively.

Cross-species comparison analysis

The scRNA-seq data of human and mouse retina was collected from two published studies (Heng et al., 2019; Lukowski et al., 2019). For comparison analysis between human being and other species, only six cell types were used, including Rod, Cone, Bipolar, Müller, MG and RGC. The cell type information was directly collected from the corresponding studies. Then, 19,450 and 12,832 single cells in human and mouse were obtained for the downstream analysis, respectively. Only the homologous genes between human and mouse was used, and genes with one to many correspondences were also discarded. Gene expression level was quantified with TPM in these three datasets (monkey, human and mouse), and was transformed into $\log_2(TPM/10 + 1)$.

Cross-species comparison analysis was performed with the method as same as previous studies (Cui et al., 2019; La Manno et al., 2016). We firstly identified DEGs among selected cell types in human and mouse with function *FindAllMarkers* (a standard AUC classifier was used) in R package *Seurat*, and DEGs were chosen only if the average difference of the $\log_2$-transformed TPM was greater than 0.5 with a statistical significance *power* greater than 0.25. Then, 774, 945 and 1,791 DEGs were selected in human, mouse, and monkey scRNA-seq. We calculated the average expression level of these DEGs in each cell type of each species, which was utilized in the downstream analysis. We then labelled each gene based on the coefficient of variation (CV) value, in which gene was labelled with 1 if the CV ranged from the 25th percentile to the 75th percentile and otherwise gene was labelled with 0. And therefore, we can exclude the influence of housekeeping genes and 'noisy' genes. These labeled genes make up a new sample as a control, and we calculated the coefficient of correlation between each average sample and control, and then normalized each dataset with that coefficient of correlation. Finally, we obtained the correlation result between any two average samples.

Aging-associated transcriptional variation analysis

For cells in a given cell type, aging-associated CV was defined to measure the consistency of gene expression patterns between cells collected from young monkeys and cells from aged monkeys, and this definition reflected the extent of influence on the transcriptome driven by aging, which was described similarly as in previous studies (Salzer et al., 2018; Wang et al., 2020).

As we identified cell types in all filtered cells, 1,126 HVGs were firstly selected based on the average expression and dispersion, and these HVGs were used for the downstream aging-associated transcriptional variation analysis. For each HVG g in a given cell type c (including num_y cells collected from young monkeys and num_o cells from aged monkeys), g_y and g_o were donated as the expression level in young and aged monkeys, respectively; and cell pairwise distance d_g was defined as:

$$d_g = |g_y - g_o|, \text{where } y \in \{1,2,\ldots,num_y\}, o \in \{1,2,\ldots,num_o\}.$$

Arithmetic mean of d_g was denoted as μ_g and standard deviation of d_g was denoted as σ_g, and therefore aging-associated CV was calculated as standard deviation divided by arithmetic mean for each HVG.

Identification of aging-associated differentially expressed genes

To identify aging-associated DEGs between cells collected from young monkeys and cells from aged monkeys in each cell type, based on the log_2-transformed TPM data, we performed the reproducibility optimized test statistic (ROTS) algorithm using R package *ROTS* (Elo et al., 2008; Seyednasrollah et al., 2016). Aging-associated DEGs were chosen only if the average difference of the log_2-transformed TPM was greater than 0.5 with a statistical significance P value less than 0.05. GO analysis of aging-associated DEGs was performed using website tool *Metascape* with default parameters (Zhou et al., 2019).

Potentially core transcriptional regulator analysis

Based on scRNA-seq data in which gene expression levels were quantified with raw UMI, co-regulation transcriptional network analysis was performed to identify the potentially core transcriptional regulators in a given cell type with given genes. To construct the upregulated network between aging transcriptional regulators and aging targets of a given cell type, upregulated aging-associated DEGs were used as input for R package *SCENIC* (Aibar et al., 2017; Elo et al., 2008). Since there is no well-established transcriptional regulator database for *Macaca fascicularis,* whose genome highly similar to that of humans, we used the human transcriptional regulator database. Of 1,839 transcriptional regulators in the human database, we detected 1,579 regulators in the *Macaca fascicularis* gene set. The regulator network was then established using function *runGenie3* in *SCENIC* based on log_2-tranforrmed UMI data. Only the transcriptional regulator-target connected with a high weight (weight > 0.1) was retained for the downstream analysis. The same process was executed for the downregulated DEG network.

For visualization of the network, the node size indicates the number of connections and the line size indicates the weight of a connection; and nodes are ranked based on node size.

Cell-cell interaction analysis

Based on scRNA-seq data in which gene expression levels were quantified with raw TPM instead of log_2-transformed TPM, the cell-cell interaction analysis was performed using *CellPhoneDB* (version: 2.0.0) (Ma et al., 2020; Vento-Tormo et al., 2018; Zheng et al., 2020). For a given cell type, only the ligand and receptor interacting pair satisfying the following three criteria was considered in the downstream analysis: (1) at least 10% cells expressed that pair (set the parameter as '*--threshold 0.1*'), (2) the average expression level was greater than 5, and (3) compared between different cell types, the enrichment of the pair was statistically significant (P < 0.05). A ligand-receptor pair detected only in cells collected from young monkeys was considered as a young-specific interaction pair; a ligand-receptor pair detected only in cells from aged monkeys was considered as an aged-specific interaction pair; the rest were considered as common interaction pairs. GO analysis of newborn or disappeared interactions was performed using website tool *Metascape* with default parameters.

Network visualization of high-risk aging-associated differentially expressed genes

The eye disease-associated gene set was collected from two previous studies (Elo et al., 2008; Orozco et al., 2020). Given that several of the same eye diseases were included in these two studies, such as 'retinitis pigmentosa', 'age-related macular degeneration', 'macular degeneration' and 'congenital stationary night blindness', we merged genes in the same eye diseases in our downstream analysis. GenAge gene set of aging/longevity-related genes was collected from public website (Magalhães et al., 2009): https://www.genomics.senescence.info/.

Statistical analysis

All data were statistically analyzed using PRISM software (GraphPad 6 Software). Results were presented as mean ± SEM. Comparisons were conducted using the one-tailed or two-tailed Student's *t*-test as indicated in figure legend.
The bioinformatics data were statistically analyzed using a two-tailed Student's *t*-test with R language, and *P* values are indicated in each figure.

Data availability

All RNA-seq raw sequencing data and processed data have been deposited in the NCBI Gene Expression Omnibus (GEO) under the accession number GSE113917.

Reference

Aibar, S., González-Blas, C.B., Moerman, T., Huynh-Thu, V.A., Imrichova, H., Hulselmans, G., Rambow, F., Marine, J.-C., Geurts, P., Aerts, J., *et al.* (2017). SCENIC: single-cell regulatory network inference and clustering. nature methods *14*, 1083-1086.

Anders, S., Pyl, P.T., and Huber, W. (2015). HTSeq-a python framework to work with high-throughput sequencing data. Bioinformatics *31*, 166–169.

Chen, J., Bardes, E.E., Aronow, B.J., and Jegga, A.G. (2009). ToppGene Suite for gene list enrichment analysis and candidate gene prioritization. Nucleic acids research *37*, W305-311.

Cui, Y., Zheng, Y., Liu, X., Yan, L., Fan, X., Yong, J., Hu, Y., Dong, J., Li, Q., Wu, X., *et al.* (2019). Single-Cell Transcriptome Analysis Maps the Developmental Track of the Human Heart. Cell Rep *26*, 1934-1950 e1935.

Dong, J., Hu, Y., Fan, X., Wu, X., Mao, Y., Hu, B., Guo, H., Wen, L., and Tang, F. (2018). Single-cell RNA-seq analysis unveils a prevalent epithelial/mesenchymal hybrid state during mouse organogenesis. Genome biology *19*, 31.

Dorey, C.K., Wu, G., Ebenstein, D., Garsd, A., and Weiter, J.J. (1989). Cell loss in the aging retina. Relationship to lipofuscin accumulation and macular degeneration. Invest Ophthalmol Vis Sci *30*, 1691-1699.

Elo, L.L., Filen, S., Lahesmaa, R., and Aittokallio, T. (2008). Reproducibility-Optimized Test Statistic for Ranking Genes in Microarray Studies. ieee acm transactions on computational biology and bioinformatics *5*, 423-431.

Haber, A.L., Biton, M., Rogel, N., Herbst, R.H., Shekhar, K., Smillie, C., Burgin, G., Delorey, T.M., Howitt, M.R., Katz, Y., *et al.* (2017). A single-cell survey of the small intestinal epithelium. Nature *551*, 333-339.

Heng, J.S., Rattner, A., Stein-O'Brien, G.L., Winer, B.L., Jones, B.W., Vernon, H.J., Goff, L.A., and Nathans, J. (2019). Hypoxia tolerance in the Norrin-deficient retina and the chronically hypoxic brain studied at single-cell resolution. Proceedings of the National Academy of Sciences of the

United States of America *116*, 9103-9114.
Islam, S., Kjallquist, U., Moliner, A., Zajac, P., Fan, J.B., Lonnerberg, P., and Linnarsson, S. (2012). Highly multiplexed and strand-specific single-cell RNA 5' end sequencing. Nat Protoc *7*, 813-828.
La Manno, G., Gyllborg, D., Codeluppi, S., Nishimura, K., Salto, C., Zeisel, A., Borm, L.E., Stott, S.R.W., Toledo, E.M., Villaescusa, J.C.*, et al.* (2016). Molecular Diversity of Midbrain Development in Mouse, Human, and Stem Cells. Cell *167*, 566-580 e519.
Li, J., Zheng, Y., Yan, P., Song, M., Wang, S., Sun, L., Liu, Z., Ma, S., Belmonte, J.C.I., Chan, P.*, et al.* (2020). A Single-cell Transcriptomic Atlas of Primate Pancreatic Islet Aging. National Science Review.
Li, X., Ji, G., Chen, X., Ding, W., Sun, L., Xu, W., Han, H., and Sun, F. (2017). Large scale three-dimensional reconstruction of an entire Caenorhabditis elegans larva using AutoCUTS-SEM. J Struct Biol *200*, 87-96.
Lukowski, S.W., Lo, C.Y., Sharov, A.A., Nguyen, Q., Fang, L., Hung, S.S., Zhu, L., Zhang, T., Grunert, U., Nguyen, T.*, et al.* (2019). A single-cell transcriptome atlas of the adult human retina. The EMBO journal *38*, e100811.
Ma, S., Sun, S., Geng, L., Song, M., Wang, W., Ye, Y., Ji, Q., Zou, Z., Wang, S., He, X.*, et al.* (2020). Caloric Restriction Reprograms the Single-Cell Transcriptional Landscape of Rattus Norvegicus Aging. Cell *180*, 984-1001 e1022.
Magalhães, J.P.d., Budovsky, A., Lehmann, G., Costa, J., Li, Y., Fraifeld, V., and Church, G.M. (2009). The Human Ageing Genomic Resources: online databases and tools for biogerontologists. aging cell *8*, 65-72.
Nichols, S.M., Bavister, B.D., Brenner, C.A., Didier, P.J., Harrison, R.M., and Kubisch, H.M. (2005). Ovarian senescence in the rhesus monkey (*Macaca mulatta*). Hum Reprod *20*, 79–83.
Orozco, L.D., Chen, H.-H., Cox, C., Katschke, K.J., Arceo, R., Espiritu, C., Caplazi, P., Nghiem, S.S., Chen, Y.-J., Modrusan, Z.*, et al.* (2020). Integration of eQTL and a Single-Cell Atlas in the Human Eye Identifies Causal Genes for Age-Related Macular Degeneration. cell reports *30*.
Qiu, X., Hill, A., Packer, J., Lin, D., Ma, Y.-A., and Trapnell, C. (2017). Single-cell mRNA quantification and differential analysis with Census. nature methods *14*, 309-315.
Salzer, M.C., Lafzi, A., Berenguer-Llergo, A., Youssif, C., Castellanos, A., Solanas, G., Peixoto, F.O., Stephan-Otto Attolini, C., Prats, N., Aguilera, M.*, et al.* (2018). Identity Noise and Adipogenic Traits Characterize Dermal Fibroblast Aging. Cell *175*, 1575-1590 e1522.
Satija, R., Farrell, J.A., Gennert, D., Schier, A.F., and Regev, A. (2015). Spatial reconstruction of single-cell gene expression data. Nat Biotechnol *33*, 495–502.
Seyednasrollah, F., Rantanen, K., Jaakkola, P., and Elo, L.L. (2016). ROTS: reproducible RNA-seq biomarker detector—prognostic markers for clear cell renal cell cancer. nucleic acids research *44*.
Trapnell, C., Cacchiarelli, D., Grimsby, J., Pokharel, P., Li, S., Morse, M., Lennon, N.J., Livak, K.J., Mikkelsen, T.S., and Rinn, J.L. (2014). The dynamics and regulators of cell fate decisions are revealed by pseudotemporal ordering of single cells. nature biotechnology *32*, 381-386.
Trapnell, C., Pachter, L., and Salzberg, S.L. (2009). TopHat: discovering splice junctions with RNA-Seq. Bioinformatics *25*, 1105-1111.
Vento-Tormo, R., Efremova, M., Botting, R.A., Turco, M.Y., Vento-Tormo, M., Meyer, K.B., Park, J.-E., Stephenson, E., Polański, K., Goncalves, A.*, et al.* (2018). Single-cell reconstruction of the early maternal–fetal interface in humans. Nature *563*, 347-353.
Wang, S., Zheng, Y., Li, J., Yu, Y., Zhang, W., Song, M., Liu, Z., Min, Z., Hu, H., Jing, Y.*, et al.* (2020).

Single-Cell Transcriptomic Atlas of Primate Ovarian Aging. Cell *180*, 585-600 e519.
Zhang, W., Zhang, S., Yan, P., Ren, J., Song, M., Li, J., Lei, J., Pan, H., Wang, S., Ma, X., *et al.* (2020). A single-cell transcriptomic landscape of primate arterial aging. Nature communications *11*, 2202.
Zheng, Y., Liu, X., Le, W., Xie, L., Li, H., Wen, W., Wang, S., Ma, S., Huang, Z., Ye, J., *et al.* (2020). A human circulating immune cell landscape in aging and COVID-19. Protein Cell.
Zhou, Y., Zhou, B., Pache, L., Chang, M., Khodabakhshi, A.H., Tanaseichuk, O., Benner, C., and Chanda, S.K. (2019). Metascape provides a biologist-oriented resource for the analysis of systems-level datasets. Nature Communications *10*, 1523.

Supplementary Figure Legends

Figure S1. Quality control information of single-cell RNA-seq data.

(A) Table showing basic information about the cynomolgus monkey cohort.

(B) Representative immunofluorescence images of ARR3-positive Cone in young and old monkeys. Scale bar, 50 μm.

(C) Representative H&E stained images of RPE in young and old monkeys. Scale bar, 50 μm.

(D) Histograms showing the cell distribution with different mapping rate (the ratio of uniquely mapped reads) (left) or different number of genes (right) detected in each single cell.

(E) *t*-SNE plots showing the single cell distribution in individual monkeys (left) and age groups (right).

(F) Bar plots showing the expression level of representative known and novel marker genes for various cell types. All expression levels are measured using the same scale. Data are shown as mean ± SEM.

Figure S2. Subtype identification of Müller glial cells and cross-species comparisons.

(A) *t*-SNE plot showing the two subtypes of Müller glial cells.

(B) Left, heatmap showing the gene expression signatures of each Müller glial cell subtype, and the number of DEGs is shown on the left of the heatmap. Right, representative GO terms of the DEGs in each cell subtype. Values in each row are *z*-score scaled. The color key from blue to red indicates low to high gene expression levels.

(C) Trajectory analysis showing the pseudotime of Müller glial subtype cells and other cells. Arrow indicates the pseudotime from early ones to late ones.

(D) Bar plots showing gene expression signatures of representative retinal progenitor cell markers in two Müller subtypes. Two-tailed Student's *t*-test *P* values are indicated.

(E) Heatmaps showing cross-species comparison correlation between human and human (left), between human and mouse (middle), and between human and monkey (right) cells. The value in each diagonal indicates the correlation coefficient.

Figure S3. Cell identity and transcriptional noise analysis during aging process.

(A) Dot plot showing the expression level of representative marker genes across different cell types in both young (red dots) and old (blue dots) monkeys. Dot color indicates the scaled gene expression levels, and dot size indicates the percentage of expressed cells.

(B) Coefficient of variation (CV) analysis showing the transcriptional noise of different cell types.

Figure S4. Transcriptional signatures of genes in the GenAge gene set and retinal disease gene sets.

Heatmaps showing the expression level of genes in the GenAge gene set and retinal disease gene sets in different cell types. Values in each row are *z*-score scaled. The color key from blue to red indicates low to high gene expression levels.

Figure S5. Age-related transcriptional alterations in various cell types.

(A) Box plots showing SASP score in different cell types from the neural retina layer or choroid layer. Two-tailed Student's *t*-test *P* values are indicated. SASP score is calculated as the average expression level of SASP-related genes in each single cell.

(B) Dot plot showing the $\log_2$-transformed fold change of SASP-related genes between

cells from aged monkeys and cells from young monkeys. Only genes with statistically significant difference between cells from aged monkeys and cells from young monkeys are shown. Dot color indicates the log_2-transformed fold change. Genes which are only upregulated in given cell types are shown in the left (red shadow), and genes which are only downregulated in given cell types are shown in the right (blue shadow).

Figure S6. Integrative analysis of aging-associated differentially expressed genes with genes from GenAge gene set.
Heatmaps showing the overlapping genes between aging-associated DEGs and genes in the GenAge gene set. Left, upregulated aging-associated DEGs; right, downregulated aging-associated DEGs.

Figure S7. Integrative analysis of aging-associated differentially expressed genes with genes from retinal disease gene sets.
(A) Heatmaps showing the overlapping genes between aging-associated DEGs and genes in different retinal disease gene sets. Each panel corresponds to different diseases. Left, upregulated aging-associated DEGs; right, downregulated aging-associated DEGs.
(B) Heatmaps showing the overlapping genes between aging-associated DEGs and genes in the retinal disease gene set. This gene set is the union set of all different retinal diseases corresponding to Figure S7A. Top, upregulated aging-associated DEGs; bottom, downregulated aging-associated DEGs.

Figure S8. Age-related cell-cell interaction alterations between monkey RPE cells and other cells in neural retina or choroid.
Dot plots showing newborn (only existed in aged monkeys, left) and disappeared (only existed in young monkeys, right) ligand-receptor gene pairs in a given cell pair. For a given cell pair, a given gene pair is either a newborn one or a disappeared one in this figure. The dot color and size indicate the average expression level and the statistical significance of gene pairs, respectively. In each row, pink and blue words correspond to ligands and receptors, respectively; in each column, pink words correspond to cell types expressing ligands and blue words correspond to cell types expressing receptors.

Figure S9. Potentially core transcriptional regulators during aging process across different cell types.
(A) Regulatory network showing potentially core transcriptional regulators in upregulated aging-associated DEGs of RPE cells. The line thickness indicates the weight of a connection, and the dot size indicates the number of connections. Top-ranked 4 nodes (ranked by the number of connections) are colored with red. Only connections with high weights are kept.
(B) Dot plots showing potentially core transcriptional regulators in aging-associated DEGs across different cell types. The dot size indicates the number of connections. Only connections with high weights are kept.

Supplementary Table Legends

Table S1. Basic quality control information of single-cell RNA-seq dataset.

Table S2. Cell clustering, cell-type-specific marker genes and DEGs between two Müller glial subtype cells.

Table S3. SASP-related genes, the GenAge gene set and retinal disease gene sets.

Table S4. Aging-associated DEGs and corresponding GO terms in each cell type.

Table S5. Cell-cell interaction information between any two cell types.

Table S6. Antibodies used in this study.

Figure S1

A

No.	Animal symbol	Sex	Age (year)	Number of total cells	Number of cells after filtering
1	YF1	Female	~ 5	412	242
2	YF2	Female	~ 5	472	438
3	YF3	Female	~ 5	479	441
4	YF4	Female	~ 4	473	408
5	YM1	Male	~ 5	480	423
6	YM2	Male	~ 5	430	360
7	YM3	Male	~ 6	447	390
8	YM4	Male	~ 6	480	363
9	OF1	Female	~ 18	480	443
10	OF2	Female	~ 19	460	410
11	OF3	Female	~ 19	480	412
12	OF4	Female	~ 20	480	440
13	OM1	Male	~ 18	455	357
14	OM2	Male	~ 19	475	401
15	OM3	Male	~ 20	479	443
16	OM4	Male	~ 21	479	439

B

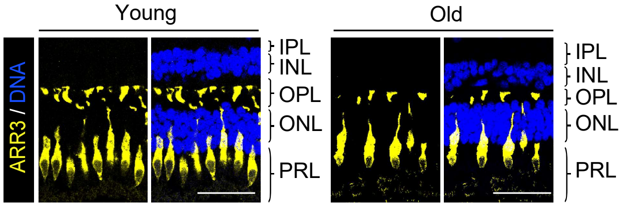

C

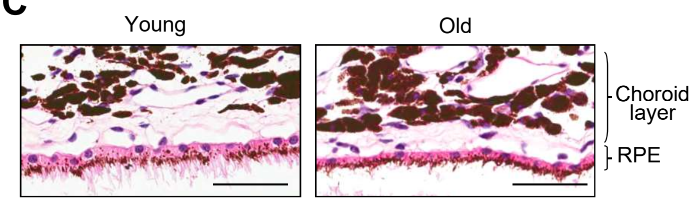

D

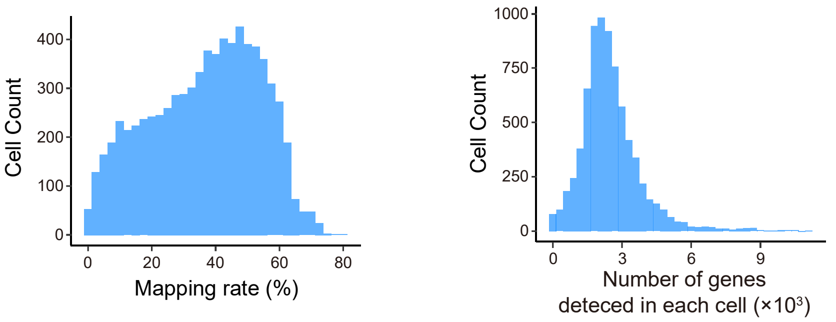

E

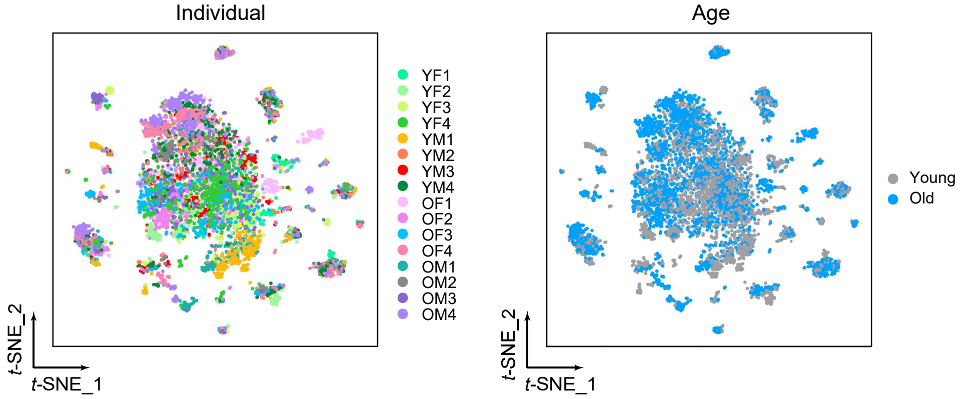

F

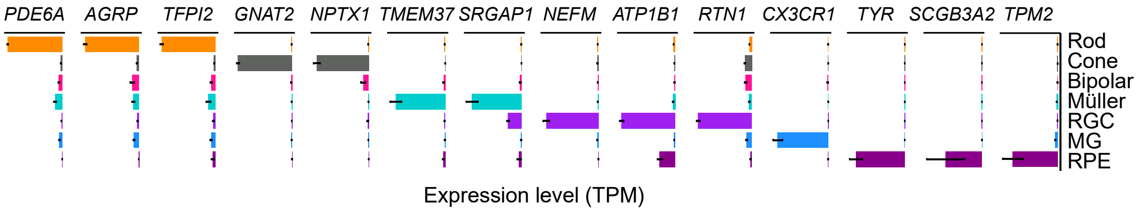

Figure S2

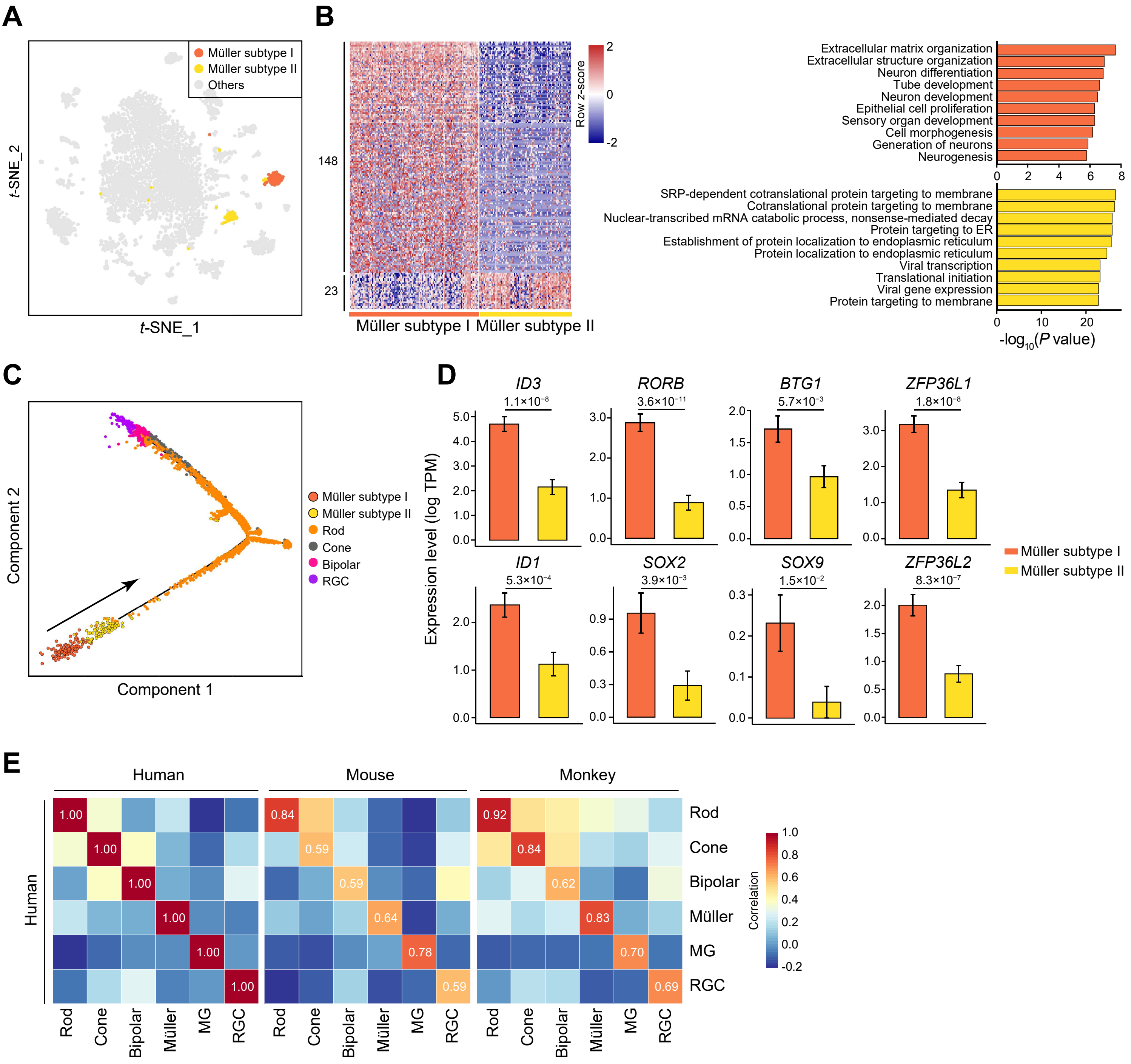
A
B
C
D
E
Müller subtype I
Müller subtype II
Others
t-SNE_2
t-SNE_1
148
23
Row z-score
2
0
-2
Müller subtype I Müller subtype II
Extracellular matrix organization
Extracellular structure organization
Neuron differentiation
Tube development
Neuron development
Epithelial cell proliferation
Sensory organ development
Cell morphogenesis
Generation of neurons
Neurogenesis
SRP-dependent cotranslational protein targeting to membrane
Cotranslational protein targeting to membrane
Nuclear-transcribed mRNA catabolic process, nonsense-mediated decay
Protein targeting to ER
Establishment of protein localization to endoplasmic reticulum
Protein localization to endoplasmic reticulum
Viral transcription
Translational initiation
Viral gene expression
Protein targeting to membrane
-log10(P value)
Component 2
Component 1
Müller subtype I
Müller subtype II
Rod
Cone
Bipolar
RGC
Expression level (log TPM)
ID3
1.1×10⁻⁸
RORB
3.6×10⁻¹¹
BTG1
5.7×10⁻³
ZFP36L1
1.8×10⁻⁸
ID1
5.3×10⁻⁴
SOX2
3.9×10⁻³
SOX9
1.5×10⁻²
ZFP36L2
8.3×10⁻⁷
Müller subtype I
Müller subtype II
Human
Mouse
Monkey
Human
Rod
Cone
Bipolar
Müller
MG
RGC
Correlation
1.00
0.84
0.92
0.59
0.84
0.59
0.62
0.64
0.83
0.78
0.70
0.59
0.69

Figure S3

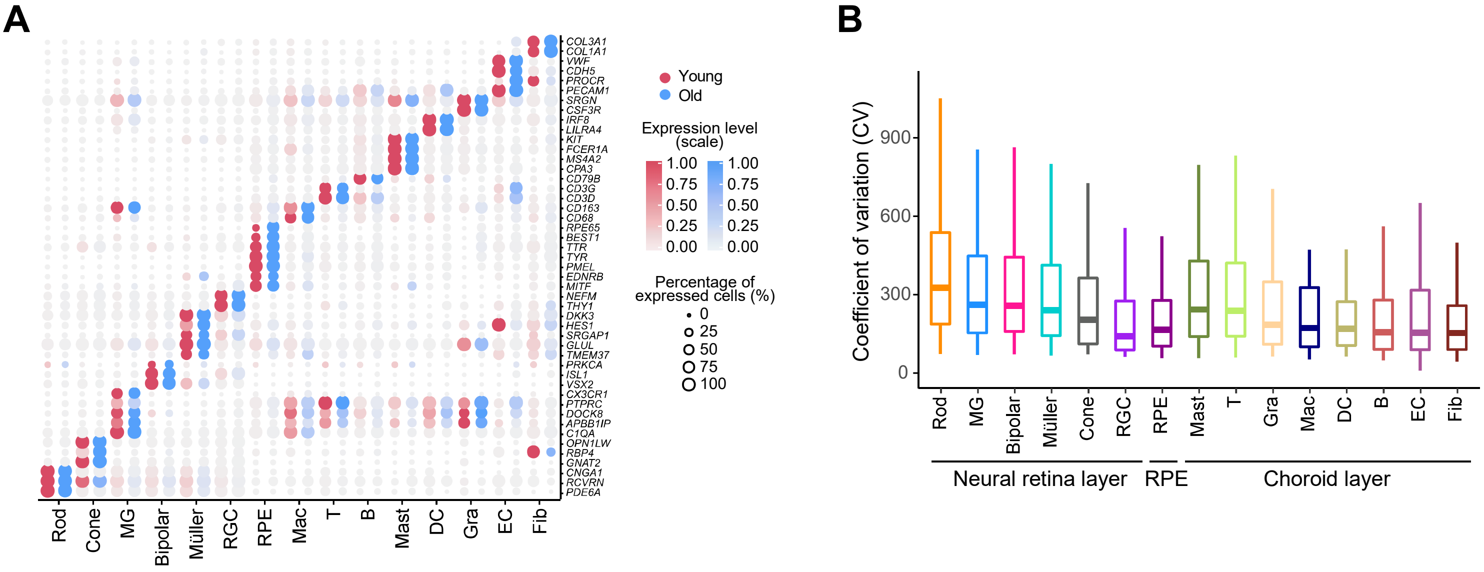
A
B
Young
Old
Expression level (scale)
1.00
0.75
0.50
0.25
0.00
Percentage of expressed cells (%)
0
25
50
75
100
COL3A1
COL1A1
VWF
CDH5
PROCR
PECAM1
SRGN
CSF3R
IRF8
LILRA4
KIT
FCER1A
MS4A2
CPA3
CD79B
CD3G
CD3D
CD163
CD68
RPE65
BEST1
TTR
TYR
PMEL
EDNRB
MITF
NEFM
THY1
DKK3
HES1
SRGAP1
GLUL
TMEM37
PRKCA
ISL1
VSX2
CX3CR1
PTPRC
DOCK8
APBB1IP
C1QA
OPN1LW
RBP4
GNAT2
CNGA1
RCVRN
PDE6A
Rod
Cone
MG
Bipolar
Müller
RGC
RPE
Mac
T
B
Mast
DC
Gra
EC
Fib
Coefficient of variation (CV)
900
600
300
0
Neural retina layer
RPE
Choroid layer

Figure S4

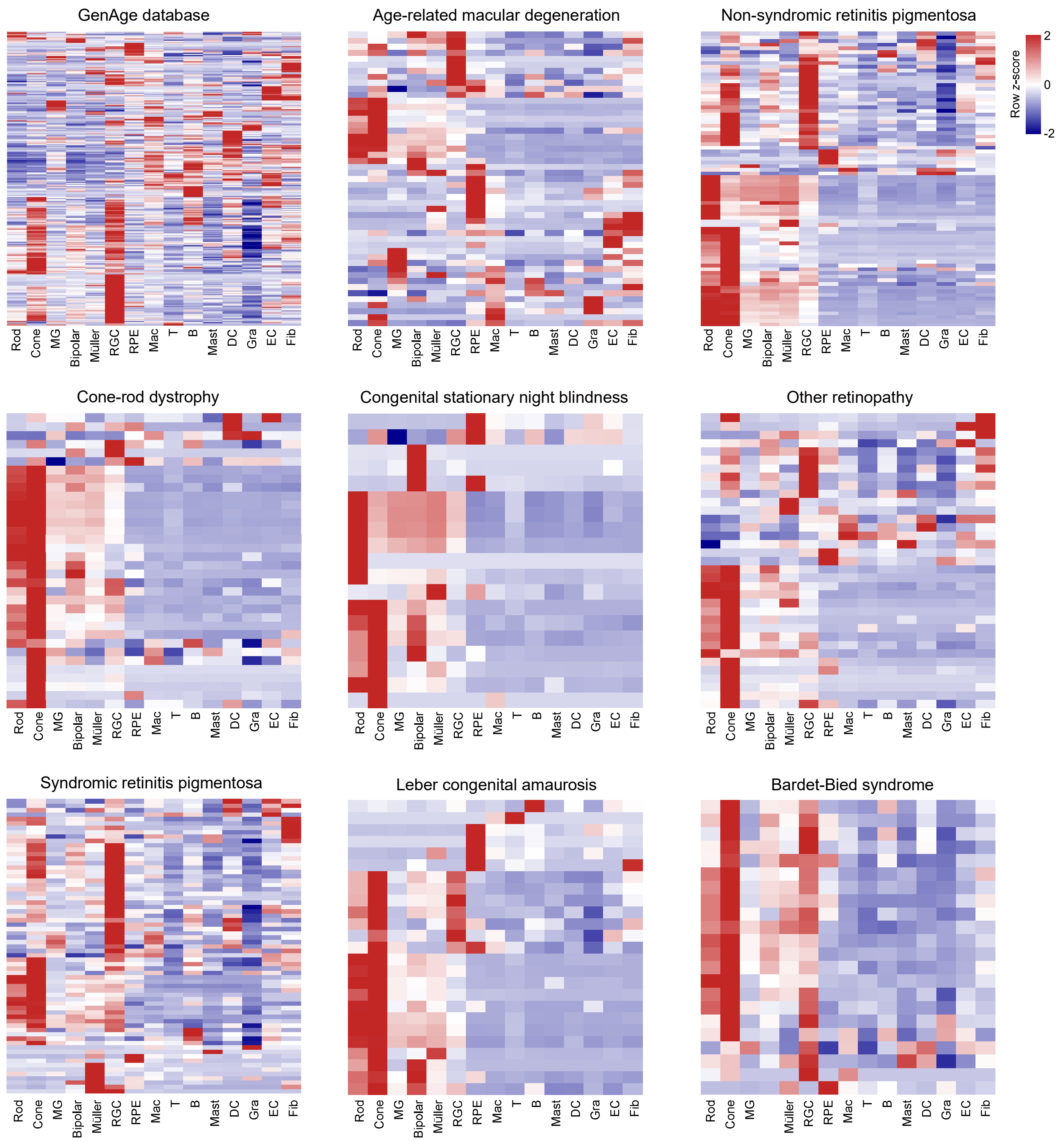

GenAge database
Age-related macular degeneration
Non-syndromic retinitis pigmentosa
Cone-rod dystrophy
Congenital stationary night blindness
Other retinopathy
Syndromic retinitis pigmentosa
Leber congenital amaurosis
Bardet-Bied syndrome
Rod
Cone
MG
Bipolar
Müller
RGC
RPE
Mac
T
B
Mast
DC
Gra
EC
Fib
Row z-score
2
0
-2

Figure S5

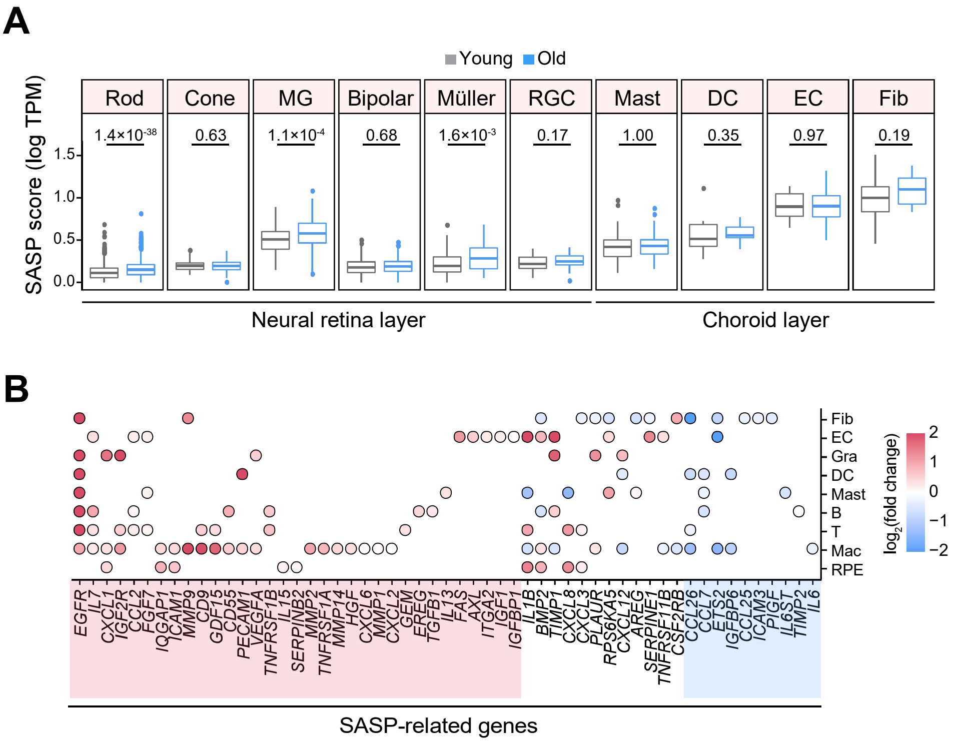
A
Young
Old
SASP score (log TPM)
Rod
Cone
MG
Bipolar
Müller
RGC
Mast
DC
EC
Fib
1.4×10-38
0.63
1.1×10-4
0.68
1.6×10-3
0.17
1.00
0.35
0.97
0.19
Neural retina layer
Choroid layer
B
Fib
EC
Gra
DC
Mast
B
T
Mac
RPE
log2(fold change)
EGFR IL7 CXCL1 IGF2R CCL2 FGF7 IQGAP1 ICAM1 MMP9 CD9 GDF15 CD55 PECAM1 VEGFA TNFRSF1B IL15 SERPINB2 MMP2 TNFRSF1A MMP14 HGF CXCL6 MMP1 CXCL2 GEM EREG TGFB1 IL13 FAS AXL ITGA2 IGF1 IGFBP1 IL1B BMP2 TIMP1 CXCL8 CXCL3 PLAUR RPS6KA5 CXCL12 AREG SERPINE1 TNFRSF11B CSF2RB CCL26 CCL7 ETS2 IGFBP6 CCL25 ICAM3 PIGF IL6ST TIMP2 IL6
SASP-related genes

Figure S6

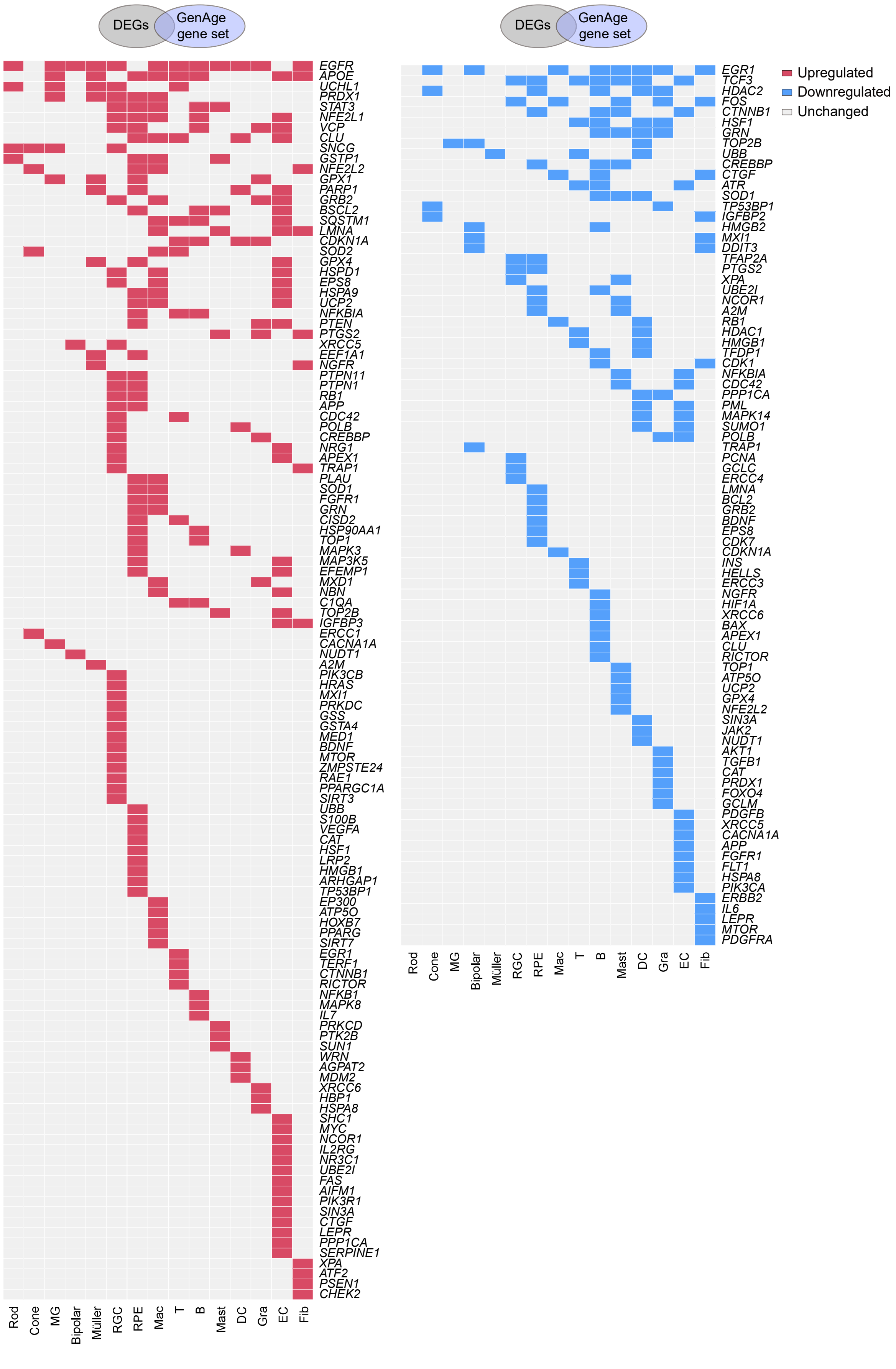
DEGs
GenAge gene set
EGFR
APOE
UCHL1
PRDX1
STAT3
NFE2L1
VCP
CLU
SNCG
GSTP1
NFE2L2
GPX1
PARP1
GRB2
BSCL2
SQSTM1
LMNA
CDKN1A
SOD2
GPX4
HSPD1
EPS8
HSPA9
UCP2
NFKBIA
PTEN
PTGS2
XRCC5
EEF1A1
NGFR
PTPN11
PTPN1
RB1
APP
CDC42
POLB
CREBBP
NRG1
APEX1
TRAP1
PLAU
SOD1
FGFR1
GRN
CISD2
HSP90AA1
TOP1
MAPK3
MAP3K5
EFEMP1
MXD1
NBN
C1QA
TOP2B
IGFBP3
ERCC1
CACNA1A
NUDT1
A2M
PIK3CB
HRAS
MXI1
PRKDC
GSS
GSTA4
MED1
BDNF
MTOR
ZMPSTE24
RAE1
PPARGC1A
SIRT3
UBB
S100B
VEGFA
CAT
HSF1
LRP2
HMGB1
ARHGAP1
TP53BP1
EP300
ATP5O
HOXB7
PPARG
SIRT7
EGR1
TERF1
CTNNB1
RICTOR
NFKB1
MAPK8
IL7
PRKCD
PTK2B
SUN1
WRN
AGPAT2
MDM2
XRCC6
HBP1
HSPA8
SHC1
MYC
NCOR1
IL2RG
NR3C1
UBE2I
FAS
AIFM1
PIK3R1
SIN3A
CTGF
LEPR
PPP1CA
SERPINE1
XPA
ATF2
PSEN1
CHEK2
Rod
Cone
MG
Bipolar
Müller
RGC
RPE
Mac
T
B
Mast
DC
Gra
EC
Fib
DEGs
GenAge gene set
EGR1
TCF3
HDAC2
FOS
CTNNB1
HSF1
GRN
TOP2B
UBB
CREBBP
CTGF
ATR
SOD1
TP53BP1
IGFBP2
HMGB2
MXI1
DDIT3
TFAP2A
PTGS2
XPA
UBE2I
NCOR1
A2M
RB1
HDAC1
HMGB1
TFDP1
CDK1
NFKBIA
CDC42
PPP1CA
PML
MAPK14
SUMO1
POLB
TRAP1
PCNA
GCLC
ERCC4
LMNA
BCL2
GRB2
BDNF
EPS8
CDK7
CDKN1A
INS
HELLS
ERCC3
NGFR
HIF1A
XRCC6
BAX
APEX1
CLU
RICTOR
TOP1
ATP5O
UCP2
GPX4
NFE2L2
SIN3A
JAK2
NUDT1
AKT1
TGFB1
CAT
PRDX1
FOXO4
GCLM
PDGFB
XRCC5
CACNA1A
APP
FGFR1
FLT1
HSPA8
PIK3CA
ERBB2
IL6
LEPR
MTOR
PDGFRA
Rod
Cone
MG
Bipolar
Müller
RGC
RPE
Mac
T
B
Mast
DC
Gra
EC
Fib
Upregulated
Downregulated
Unchanged

Figure S7

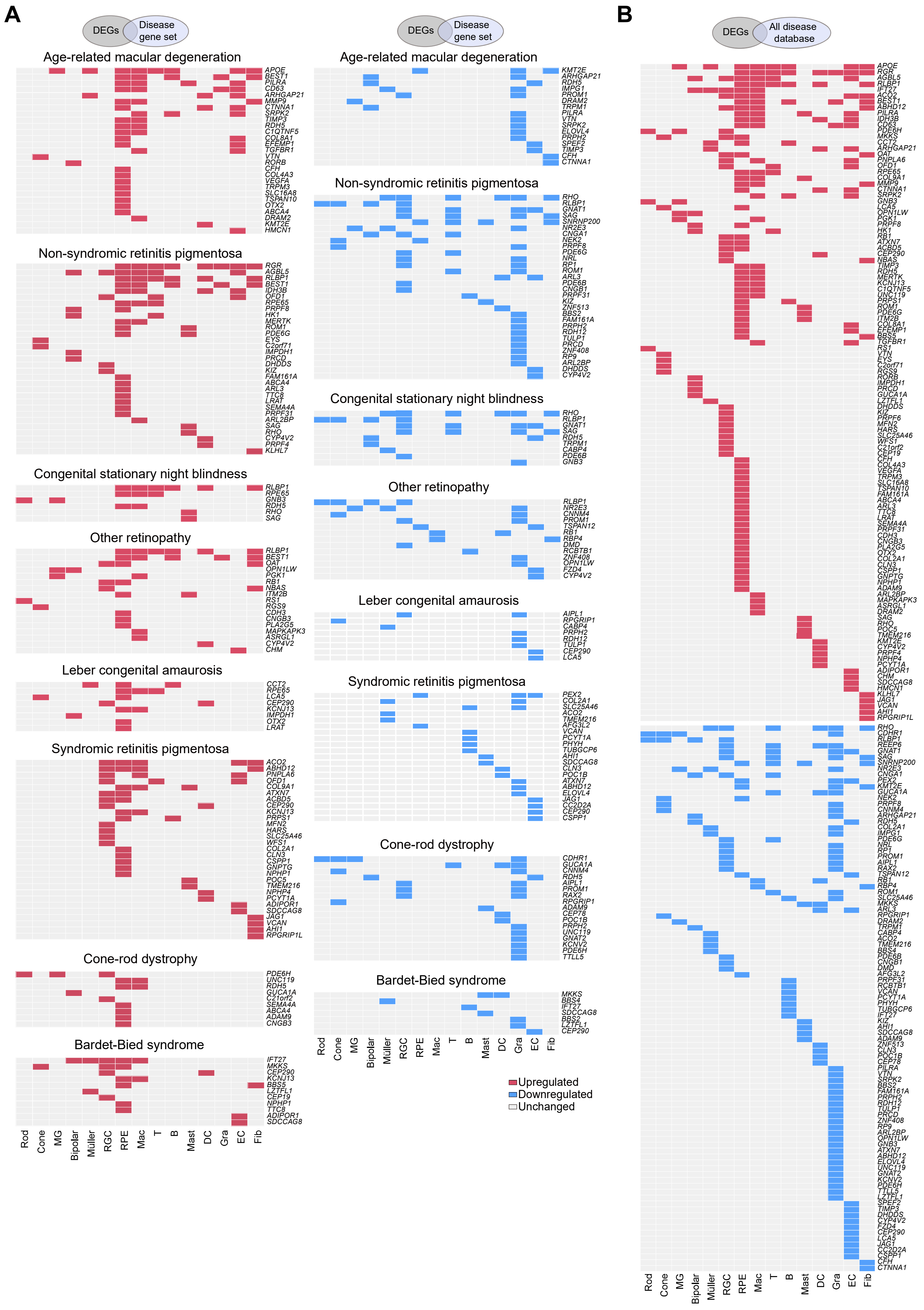
A
DEGs
Disease gene set
Age-related macular degeneration
Non-syndromic retinitis pigmentosa
Congenital stationary night blindness
Other retinopathy
Leber congenital amaurosis
Syndromic retinitis pigmentosa
Cone-rod dystrophy
Bardet-Bied syndrome
Rod
Cone
MG
Bipolar
Müller
RGC
RPE
Mac
T
B
Mast
DC
Gra
EC
Fib
Upregulated
Downregulated
Unchanged
B
DEGs
All disease database

Figure S8

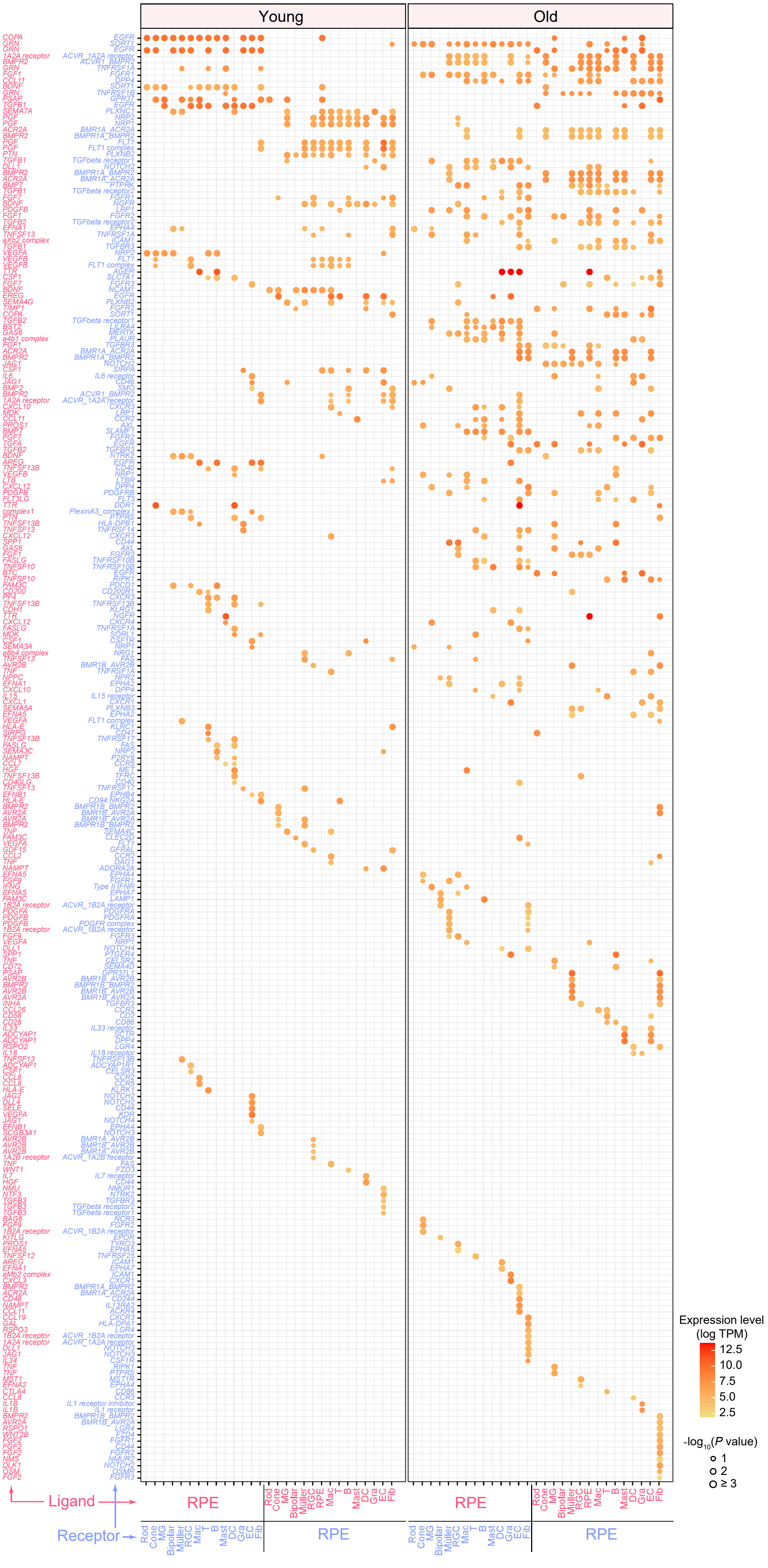
Young
Old
Expression level
(log TPM)
12.5
10.0
7.5
5.0
2.5
-log10(P value)
1
2
≥ 3
Ligand
Receptor
RPE
Rod
Cone
MG
Bipolar
Müller
RGC
Mac
T
B
Mast
DC
Gra
EC
Fib

Figure S9

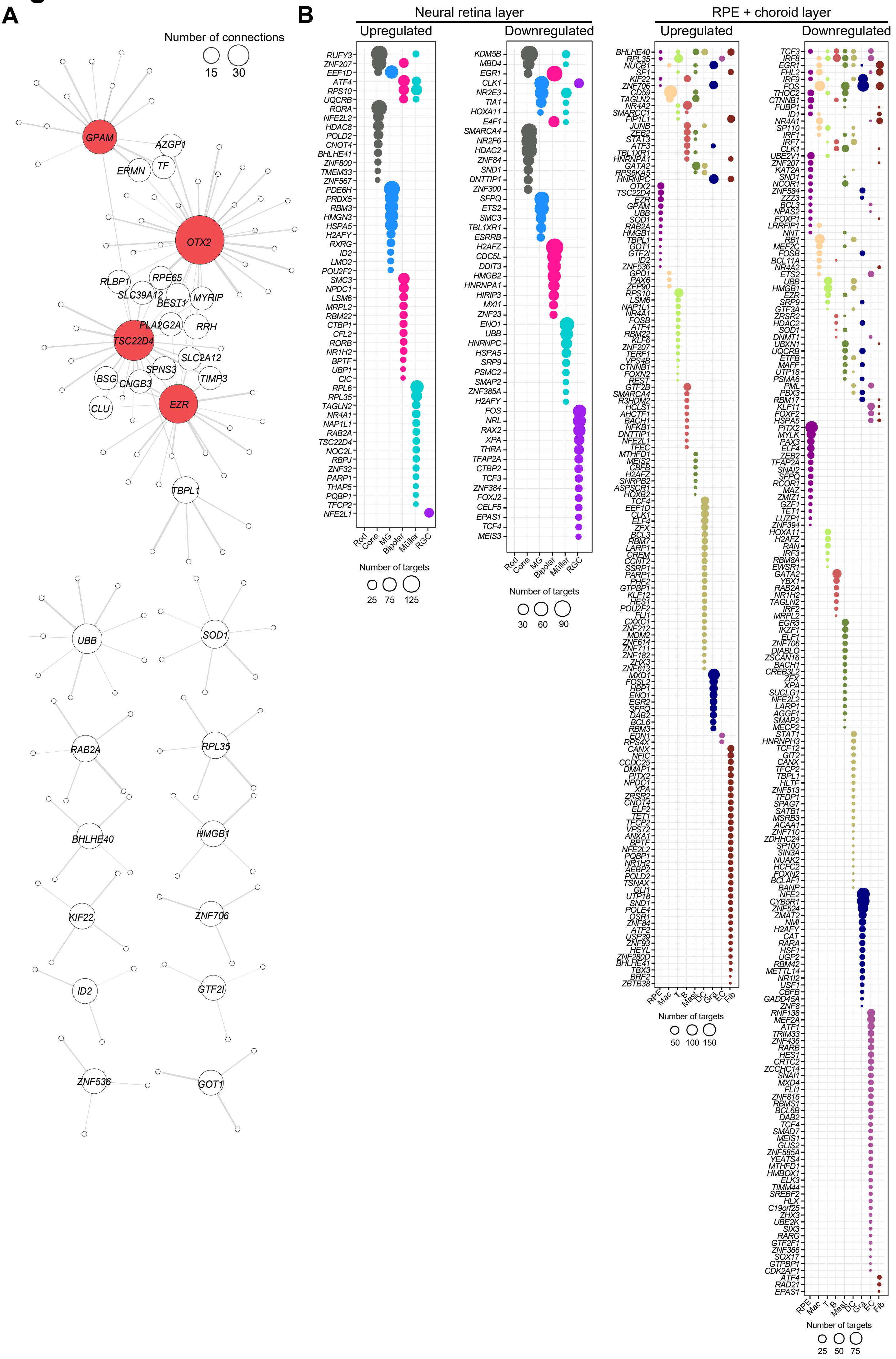
A
Number of connections
15
30
GPAM
AZGP1
ERMN
TF
OTX2
RLBP1
RPE65
SLC39A12
BEST1
MYRIP
PLA2G2A
RRH
TSC22D4
SLC2A12
SPNS3
TIMP3
BSG
CNGB3
CLU
EZR
TBPL1
UBB
SOD1
RAB2A
RPL35
BHLHE40
HMGB1
KIF22
ZNF706
ID2
GTF2I
ZNF536
GOT1
B
Neural retina layer
Upregulated
Downregulated
RPE + choroid layer
Upregulated
Downregulated
RUFY3
ZNF207
EEF1D
ATF4
RPS10
UQCRB
RORA
NFE2L2
HDAC8
POLD2
CNOT4
BHLHE41
ZNF800
TMEM33
ZNF567
PDE6H
PRDX5
RBM3
HMGN3
HSPA5
H2AFY
RXRG
ID2
LMO2
POU2F2
SMC3
NPDC1
LSM6
MRPL2
RBM22
CTBP1
CFL2
RORB
NR1H2
BPTF
UBP1
CIC
RPL6
RPL35
TAGLN2
NR4A1
NAP1L1
RAB2A
TSC22D4
NOC2L
RBPJ
ZNF32
PARP1
THAP5
PQBP1
TFCP2
NFE2L1
Rod
Cone
MG
Bipolar
Müller
RGC
Number of targets
25
75
125
KDM5B
MBD4
EGR1
CLK1
NR2E3
TIA1
HOXA11
E4F1
SMARCA4
NR2F6
HDAC2
ZNF84
SND1
DNTTIP1
ZNF300
SFPQ
ETS2
SMC3
TBL1XR1
ESRRB
H2AFZ
CDC5L
DDIT3
HMGB2
HNRNPA1
HIRIP3
MXI1
ZNF23
ENO1
UBB
HNRNPC
HSPA5
SRP9
PSMC2
SMAP2
ZNF385A
H2AFY
FOS
NRL
RAX2
XPA
THRA
TFAP2A
CTBP2
TCF3
ZNF384
FOXJ2
CELF5
EPAS1
TCF4
MEIS3
Rod
Cone
MG
Bipolar
Müller
RGC
Number of targets
30
60
90
RPE
Mac
T
B
Mast
DC
Gra
EC
Fib
Number of targets
50
100
150
RPE
Mac
T
B
Mast
DC
Gra
EC
Fib
Number of targets
25
50
75
